# Influence of Gum Arabic Enriched with GABA Coating on Oxidative Damage of Walnut Kernels

**DOI:** 10.17113/ftb.57.04.19.6380

**Published:** 2019-12

**Authors:** Asghar Ebrahimzadeh, Farhad Pirzad, Hamidreza Tahanian, Morteza Soleimani Aghdam

**Affiliations:** 1 Department of Horticultural Sciences, University of Maragheh, Madar Street, 8311155181 Maragheh, Iran; 2 Department of Horticultural Science, University College of Agriculture and Natural Resources, University of Tehran, Daneshkadeh Street, 3158777871 Karaj, Iran; 3 Shahrood Agricultural and Natural Resources Research and Education, Bastam Street, 3615799811 Semnan, Iran; 4 Department of Horticultural Science, Imam Khomeini International University, Imam Khomeini Street, 3414896818 Qazvin, Iran

**Keywords:** DPPH scavenging capacity, γ-aminobutyric acid, gum arabic coating, postharvest nutritional quality, walnut kernel

## Abstract

Because of the higher content of unsaturated fatty acids (UNSFA) and phenolics, walnut kernels are vulnerable to oxidative rancidity and browning due to unfavorable postharvest handling procedures. This study investigates the impact of gum arabic coating enriched with γ-aminobutyric acid (GABA) on oxidative rancidity and browning of kernels during storage at 20 °C. The results showed that the walnut kernels coated with gum arabic (5%) enriched with GABA (0.1 mM) exhibited lower oxidative rancidity and browning, manifested by lower peroxide value and malondialdehyde accumulation along with higher whiteness index. Moreover, kernels had higher UNSFA/SFA ratio as a response to lower lipoxygenase activity and H_2_O_2_ accumulation. The reduced oxidative browning in coated kernels was accompanied with lower polyphenol oxidase and higher phenylalanine ammonia-lyase activity leading to higher accumulation of phenolics and increased DPPH^•^ scavenging capacity. Based on our findings, gum arabic coating (5%) enriched with GABA (0.1 mM) may have a commercial potential for maintaining nutritional quality of walnut kernels.

## INTRODUCTION

Owing to higher unsaturated fatty acid (UNSFA) content, walnut kernels are nutritionally important in human diet. However, it makes walnut kernels vulnerable to oxidative rancidity under unfavorable postharvest handling (*1*). Oxidative rancidity is a result of UNSFA peroxidation by reactive oxygen species (ROS) accumulation or lipoxygenase (LOX) enzyme activity (*2*, *3*). Besides, oxidative rancidity is stimulated by a higher oxygen availability, higher temperatures and also by a long storage period. Peroxide and malondialdehyde (MDA) are the primary and secondary products of UNSFA peroxidation. Therefore, the peroxide value (PV) and MDA accumulation are often used as oxidative rancidity markers (*2*, *3*).

Under unfavorable postharvest handling procedures, in addition to oxidative rancidity, kernel browning occurs due to the oxidation of phenolics by polyphenol oxidase (PPO) (*4*). In walnuts, light kernel colour is a crucial sensory factor for the consumer preference. Oxidative rancidity and browning deteriorate the sensory and nutritional quality of walnut and cause their unpleasant off-flavour and shorten their shelf life, which is also unsafe to consumer health (*5*, *6*).

The kernel oxidative rancidity (due to high O_2_ availability) and surface browning by UNSFA peroxidation *via* ROS/LOX and phenolic oxidation by PPO are responsible for the nutritional quality deterioration and for reducing the sensory quality (*7*). Reducing the O_2_ availability by using edible coatings would be beneficial for attenuating oxidative rancidity and preventing browning of walnut kernels; resulting in safer kernels with high quality (*6*). The results obtained by Deng *et al*. (*8*) confirm that γ-aminobutyric acid (GABA), as an antioxidant, together with MDA inhibit the formation of reactive carbonyl intermediates during oxidative stress. The enrichment of edible coatings with powerful antioxidants such as GABA may help synergistically to preserve the kernel quality (*7*, *9*). Owing to the economic value and nutritional importance of walnut kernels, great efforts have been made by researchers to attenuate oxidative rancidity, maintain sensory and nutritional quality, and extend their shelf life by applying edible coatings combined with antioxidants such as soy protein coating enriched with catechin (*7*) and chitosan enriched with green tea (*10*), which now have potential commercial applications.

In this study, the impact of gum arabic coating enriched with GABA on the oxidative rancidity and browning of walnut kernels during storage at 20 °C for 18 weeks were evaluated. Research emphasis was on mass fractions of fatty acids and total phenolics during storage. Kernel colour changes and enzyme (phenylalanine ammonia-lyase (PAL), PPO and LOX) activity were also included in the measured parameters. Peroxide value (PV) and malondialdehyde (MDA) accumulation in kernels were also studied.

## MATERIALS AND METHODS

### Walnut kernels and treatments

Walnut fruits (*Juglans regia* cv. Chandler) were harvested from fully mature walnut orchard from Karaj, Alborz province, Iran. After the harvest, the fruits were immediately carried to the postharvest physiology of horticultural crops laboratory at the University of Tehran, Karaj, Iran. Then, the green husk and the hard shell of walnut fruits were manually removed. In a preliminary experiment, unshelled nuts, without being dried, were coated with 0, 5, 10, 15 and 20% (*m*/*V*) gum arabic alone or enriched with γ-aminobutyric acid (GABA) at a concentration of 0, 0.01, 0.1, 1 and 10 mM. Gum arabic solution was prepared according to Ali *et al*. (*11*) by dissolving 0, 5, 10, 15 and 20 g of gum arabic powder in 100 mL distilled water. The solutions were stirred at low heat (40 °C) for 60 min, then filtered to remove any undissolved impurities. The pH of the solutions was adjusted to 5.6 by 1 M NaOH.

GABA and gum arabic powder, food grade (kibble size: KB 120, *i.e.* <125 micron), was supplied by Labortecnic SA (Almeria, Spain). All other chemicals were purchased from Sigma-Aldrich, Merck (Madrid, Spain) and were of analytical grade. Different concentrations (0, 0.01, 0.1, 1 and 10 mM) of GABA were added to gum arabic solution according to Khaliq *et al*. (*12*). For coating treatments, healthy walnut kernels, macroscopically free from disorders and disease symptoms were placed in a mesh container and immersed in the coating solutions for 1 min. The coated walnut kernels were then dried for 3 h at room temperature and 100 g of kernel halves were kept at 20 ºC for 6, 12 and 18 weeks (*10*).

In the treated kernels, peroxide value (PV) and malondialdehyde (MDA) content (as oxidative rancidity markers) increased and kernel surface whiteness index (WI), as oxidative browning marker, decreased during storage at 20 °C for 18 weeks (data not shown). Coating resulted in the lowest kernel oxidative rancidity and browning. Based on these results, the solutions of gum arabic 5 and 10% with the addition of 0.1 and 1 mM GABA, respectively, were selected for further study. PV, MDA accumulation and WI were evaluated every 6 weeks during storage at 20 °C. Phenylalanine ammonia-lyase (PAL) and polyphenol oxidase (PPO) enzyme activity, total phenolic accumulation, DPPH^•^ scavenging capacity and LOX enzyme activity were also investigated.

### Kernel surface whiteness

Kernel colour was measured on the upper part of the outer surface on each half by chromatometer (CR-400; Minolta, Tokyo, Japan) using CIE colour parameters L*, a* and b*, and WI was calculated according to the following equation (*13*):

WI=100−[(100−L*)^2^+a*^2^+b*^2^]^1/2^ /1/

### Kernel PV and MDA content

Lipid extraction was measured according to Lee *et al*. (*14*). PV was measured by iodometric titration with 0.01 N Na_2_S_2_O_3_ according to AOAC method No. 965.33 (*15*) using 1 g of lipids separated from walnut kernels and expressed in mmol peroxide per kg kernel oil. MDA content was measured by the thiobarbituric acid (TBA) method described by Nepote *et al.* (*16*) and expressed in nmol TBA per g dry mass.

### Total phenolic content and DPPH• scavenging capacity

Total phenolic content was determined according to the Folin-Ciocalteu procedure (*17*). The absorbance of the samples was measured by UV/Vis spectrophotometer (PC-1650; Shimadzu Kyoto, Japan) and the content was expressed in mg gallic acid equivalents (GAE) per g dry mass (dm). Free radical DPPH^•^ scavenging activity was traced according to Brand-Williams *et al*. (*18*). The reduction percentage of DPPH^•^ was calculated according to the following equation:

Inhibition of DPPH^•^=[(*A*_control_-*A*_sample_)/*A*_control_]·100 /2/

where *A*_control_ is the absorbance of DPPH^•^ solution without extract.

### Fatty acid mass fraction

Fatty acids were quantified by forming fatty acid methyl esters according to Arena *et al*. (*19*), using gas chromatograph equipped with flame ionization detector (GC-FID model 6890 N; Agilent Technologies, Santa Clara, CA, USA). Fatty acid content was expressed in percentage as mass fraction in oil. The unsaturated/saturated fatty acid ratio was calculated by the formula:

UNSFA/SFA=(*w*(18:1)+*w*(18:2)+*w*(18:3))/(*w*(16:0)+*w*(18:0)) /3/

where according to C:D number 16:0 is palmitic, 18:0 stearic, 18:1 oleic, 18:2 linoleic and 18:3 linolenic acid.

### PAL and PPO activities

PAL was assayed based on cinnamic acid production according to Nguyen *et al.* (*20*), and was expressed in μmol cinnamic acid per mg protein per h. PPO was assayed by measuring the oxidation of catechol as substrate according to Nguyen *et al.* (*20*), and was expressed in U/mg protein.

### LOX activity and H_2_O_2_ content

LOX activity was measured by linoleic acid as substrate according to Phetsirikoon *et al.* (*21*), and was expressed in U/mg protein. The H_2_O_2_ content was measured according to Patterson *et al.* (*22*) and expressed in μmol/g dm.

### Statistical analysis

The experiment was conducted as split plot in time based on completely randomized design with three replications (100 g of kernels for each replication). Data analysis was performed using IBM SPSS software v. 21 (*23*). Differences were assessed by Tukey's test at p<0.05.

## RESULTS AND DISCUSSION

### Oxidative rancidity and browning

During storage at 20 °C for 18 weeks, we recorded a significant increase in PV and MDA accumulation and a decrease in WI of kernels (Table 1), indicating oxidative rancidity and browning incidence manifested in kernels by irreversible UNSFA peroxidation *via* ROS/LOX and phenolic oxidation by PPO. Kernels coated with gum arabic (5%) enriched with GABA (0.1 mM) exhibited lower PV and MDA accumulation and higher WI than other treatments during storage at 20 °C for 18 weeks (p<0.01; Table 1). Coating with 5% gum arabic containing 0.1 mM GABA resulted in the lowest kernel oxidative rancidity and browning. Thus, it can be clearly indicated that gum arabic coating enriched with GABA could strongly protect kernel UNSFA peroxidation and phenolic oxidation, attenuating kernel oxidative rancidity and browning.

**Table 1:** Oxidative rancidity and browning of walnut kernels coated with gum arabic enriched with γ-aminobutyric acid (GABA) during storage at 20 °C for 18 weeks

					Oxidative rancidity and browning markers		
*w*(gum arabic)/%	*c*(GABA)/mM			*t*/week	WI	PV/(mmol/kg)	*b*(MDA)/(nmol/g)	
				0	(54.3±0.3)	(0.7±0.1)	(8.3±1.3)	
0	0			6	(52.3±0.9)^abc^	(1.3±0.1)^e^	(11.3±1.2)^d^	
				12	(48±0.6)^d^	(2.6±0.1)^b^	(14.8±1.2)^b^	
				18	(41.7±0.9)^e^	(4.1±0.2)^a^	(16.1±0.5)^a^	
0	1			6	(50.3±0.3)^cd^	(0.8±0.1)^f^	(10.3±1.1)^e^	
				12	(48.7±0.4)^d^	(2.0±0.)^d^	(13.1±0.8)^c^	
				18	(45±0.7)^e^	(2.8±0.1)^b^	(15.9±1.1)^b^	
10	0			6	(53.7±0.4)^ab^	(0.8±0.01)^f^	(10.4±1.3)^e^	
				12	(50.5±0.5)^bcd^	(2.1±0.2)^c^	(12.6±0.5)^d^	
				18	(48±0.6)^d^	(2.9±0.2)^b^	(14.3±1.2)^b^	
5	0.1			6	(54±0.6)^a^	(0.7±0.2)^f^	(9.2±1.1)^f^	
				12	(52.3±0.9)^ab^	(1.8±0.2)^d^	(11.7±1.1)^d^	
				18	(50±0.5)^c^	(2.3±0.1)^c^	(14.0±0.6)^c^	
Significant				df				
Time				2	**	*	**	
Treatment				3	**	**	**	
T × T				6	**	**	**	
CV				-	2.35	8.80	8.06	

Data are presented as mean value±standard error of three replications. Different letters indicate significant differences at p=0.05. * and **Significance at p=0.05 and 0.01, respectively. WI=whiteness index, PV=peroxide value per kernel oil mass, MDA=malondialdehyde expressed as thiobarbituric acid per dry mass

### Fatty acid profile, LOX activity and H_2_O_2_ accumulation

As shown in Table 2, linoleic, linolenic and oleic acid contents decreased during storage at 20 °C for 18 weeks, while palmitic and stearic acid contents increased. As a consequence, unsaturated/saturated fatty acid (UNSFA/SFA) ratio decreased. Meanwhile, coated kernels with lower palmitic and stearic acid contents (p<0.01) and higher linoleic, linolenic and oleic acid contents (p<0.01) had higher UNSFA/SFA ratio during storage at 20 °C for 18 weeks (Table 2). Furthermore, kernels coated with 5% gum arabic and 0.1 mM GABA had lower LOX activity (p<0.01) and H_2_O_2_ accumulation (p<0.01) during storage (Table 3). Oxidative rancidity of walnut kernels is a consequence of the peroxidation of UNSFA by ROS/LOX, and a decline in UNSFA/SFA ratio was concurrent with the higher PV and MDA accumulation, as oxidative rancidity markers. In addition to the oxidation of kernels, UNSFA and LOX can participate in O_2_^•−^ accumulation (*24*). Therefore, overcoming the kernel oxidative rancidity is achievable by decreasing O_2_ availability to prevent ROS production using edible coatings or by scavenging ROS radicals (*7*-*10*). Kang *et al*. (*7*) reported that the walnut kernels coated with soy protein enriched with antioxidant catechin exhibited significantly lower UNSFA oxidation with the lower PV and MDA accumulation during storage at 35 ºC for 21 days, which was the result of the synergistic action of O_2_ barrier function of soy protein and antioxidant activity of catechin. The authors suggested that the edible soy protein coating enriched with catechin delayed UNSFA peroxidation of walnut kernels by protecting them from O_2_ exposure during storage. Another study carried out by Vidrih *et al.* (*25*) showed that the unstable linolenic acid content decreased due to its oxidation during storage, while the other fatty acids remained largely unaffected. Sabaghi *et al*. (*10*) reported that walnut kernels coated with chitosan enriched with antioxidant green tea exhibited a lower UNSFA peroxidation, confirmed by lower PV and MDA accumulation and higher sensory quality observable in lower kernel surface browning during storage at room temperature for 18 weeks, which was a consequence of the synergistic action of O_2_ barrier function of chitosan and the antioxidant activity of green tea. Christopoulos and Tsantili (*3*) reported that during storage at 20 ºC for 9 months, walnut kernel UNSFA/SFA ratio decreased and the oleic, linoleic and linolenic acid peroxidation resulted in kernel rancidity assayed by the higher PV, as oxidative kernel rancidity marker. Additionally, they reported that walnut kernels stored at low temperature (1 ºC) and packaged under N_2_ or CO_2_ exhibited lower PV, as oxidative kernel rancidity marker, which may be a consequence of the lower oleic, linoleic and linolenic acid peroxidation, resulting in higher kernel UNSFA/SFA ratio. Our results suggest that the debilitation of oxidative rancidity in walnut kernels coated with gum arabic (5%) enriched with GABA (0.1 mM) may be caused by a lower LOX enzyme activity and lower H_2_O_2_ accumulation. Eventually, higher UNSFA/SFA ratio may be an outcome of the synergistic action of gum arabic, which lowers O_2_ availability, with GABA, which scavenges the ROS (*26*). This suggests that the gum arabic coatings enriched by GABA delayed lipid oxidation of walnuts by protecting them from oxygen exposure during storage period.

**Table 2:** Fatty acid mass fraction in walnut kernels coated with gum arabic enriched with γ-aminobutyric acid (GABA) during storage at 20 °C for 18 weeks

						*w*(fatty acid)/%						
*w*(gum arabic)/%	*c*(GABA)/mM	*t*/week		Palmitic			Stearic	Oleic	Linoleic	Linolenic	UNSFA/SFA
		0		(8.0±0.4)			(2.8±0.6)	(22.9±0.5)	(52.3±0.5	(13.0±0.7)	(8.7±0.1)
0	0	6		(9.4±0.7)^d^			(3.8±0.1)^e^	(21.2±0.2)^c^	(51.5±1.2)^b^	(11.3±0.4)^e^	(6.4±0.1)^e^
		12		(9.8±0.1)^c^			(4.1±0.2)^c^	(19.5±0.4)^f^	(46.9±1.7)^d^	(10.5±0.6)^f^	(5.5±0.1)^h^
		18		(10.3±0.7)^a^			(4.6±0.4)^a^	(17.1±0.4)^h^	(40.4±1.3)^h^	(8.5±1.1)^h^	(4.4±0.1)^j^
0	1	6		(9.4±0.2)^d^			(3.2±0.4)^g^	(21.4±0.9)^c^	(51.1±03)^b^	(12.1±0.3)^bc^	(6.7±0.1)^d^
		12		(9.7±0.4)^c^			(3.8±0.2)^de^	(19.9±0.4)^e^	(47.4±1.5)^d^	(11.3±0.9)^e^	(5.8±0.1)^g^
		18		(10.2±0.1)^b^			(4.3±0.3)^b^	(17.9±0.4)^g^	(42.3±0.5)^g^	(9.3±0.8)^g^	(4.8±0.1)^i^
10	0	6		(8.4±0.1)^g^			(3.2±0.5)^g^	(22.3±0.3)^b^	(52.8±1.2)^a^	(12.3±1.4)^b^	(7.5±0.1)^b^
		12		(9.1±0.1)^e^			(3.5±0.5)^f^	(21.4±0.8)^c^	(49.5±1.5)^c^	(11.7±1.1)^de^	(6.5±0.1)^e^
		18		(9.5±0.1)^d^			(3.9±0.4)^cd^	(20.3±1.1)^d^	(43.6±1.2)^f^	(10.5±11)^f^	(5.6±0.1)1^)^
5	0.1	6		(8.1±0.1)^h^			(2.9±0.4)^h^	(22.8±0.3)^a^	(53.0±1.3)^a^	(12.9±06)^a^	(8.1±0.1)^a^
		12		(8.8±0.1)^f^			(3.2±0.2)^g^	(22.3±0.8)^b^	(51.3±1.3)^b^	(12.2±0.7)^b^	(7.2±0.2)^c^
		18		(9.1±0.2)^e^			(3.4±0.7)^f^	(21.4±0.4)^c^	(45.4±1.2)^e^	(11.7±0.5)^cd^	(6.2±0.1)^f^
Significant		df									
Time		2		**			**	**	**	**	**
Treatment		3		**			**	**	**	**	**
T×T		6		**			**	**	**	**	*
CV		-		1.21			1.64	1.44	1.96	1.35	1.09

Data are presented as mean value±standard error of three replications. Different letters indicate significant differences at p=0.05. * and **Significance at p=0.05 and 0.01, respectively. UNSFA/SFA=unsaturated/saturated fatty acid ratio

**Table 3:** Oxidative markers of walnut kernels coated with gum arabic enriched with γ-aminobutyric acid (GABA) during storage at 20 °C for 18 weeks

					Oxidative stress markers		
*w*(gum arabic)/%		*c*(GABA)/mM		*t*/week		LOX activity/(U/mg)	*b*(H_2_O_2_)/(μmol/g)	
				0		(10.3±0.3)	(14.3±0.3)	
0		0		6		(24±1.4)^defg^	(20.2±0.4)^cde^	
				12		(36.1±1.4)^bc^	(24.7±0.4)^b^	
				18		(50.6±1.3)^a^	(31.4±0.5)^a^	
0		1		6		(20.7±0.5)^fgh^	(19.9±0.5)^cde^	
				12		(31.9±1.0)^bcd^	(22.9±1.1)^bcd^	
				18		(33.1±0.4)^bc^	(30.1±0.4)^a^	
10		0		6		(17.0±0.9)^gh^	(17.4±0.4)^ef^	
				12		(29.7±0.7)^bcde^	(25.5±1.5)^b^	
				18		(37.5±1.1)^b^	(31.5±1.2)^a^	
5		0.1		6		(12.9±1.2)^h^	(14.5±1.2)^f^	
				12		(22.8±1.1)^efg^	(19.2±0.9)^def^	
				18		(28.6±1.3)^cdef^	(23.3±1.2)^bc^	
Significant				df				
Time				2		**	**	
Treatment				3		**	**	
T×T				6		*	*	
CV				-		10.84	6.21	

Data are presented as mean value±standard error of three replications. Different letters indicate significant differences at p=0.05. * and **Significance at p=0.05 and 0.01, respectively. LOX=lypoxygenase

### Total phenolic accumulation, DPPH scavenging capacity and PAL/PPO activities

As shown in Table 4, walnut kernels coated with 5% gum arabic enriched with GABA (0.1 mM) exhibited higher total phenolic accumulation (p<0.01) and DPPH scavenging capacity (p<0.01) during storage at 20 °C for 18 weeks. Moreover, kernels had lower PPO (p<0.01) and higher phenylalanine ammonia-lyase (PAL) enzyme activity (p<0.01).

**Table 4:** Phenolic metabolism of walnut kernels coated with gum arabic enriched with γ-aminobutyric acid (GABA) during storage at 20 °C for 18 weeks

							Phenolics metabolism				
*w*(gum arabic)/%		*c*(GABA)/mM		*t*/week		*w*(total phenolics as GAE)/ (mg/g)		DPPH^•^ scavenging capacity/%	PAL activity/ (μmol/mg∙ h)	PPO activity/ (U/mg)
				0		(54.4±0.9)		(65.5±0.9)	(62.4±1.1)	(9.1±0.8)
0		0		6		(35.7±0.8)^bcd^		(52.1±0.2)^cd^	(36.8±0.8)^d^	(12.8±0.7)^ef^
				12		(26.9±0.6)^ef^		(42.1±0.3)^f^	(22.3±0.3)^g^	(28.1±1.3)^c^
				18		(19.4±1.5)^f^		(35.9±0.3)^g^	(17.9±0.2)^h^	(66.1±1.3)^a^
0		1		6		(41.0±1.9)^b^		(55.7±0.3)^b^	(41.9±0.3)^c^	(11.0±1.8)^f^
				12		(33.0±1.8)^cde^		(49.2±0.3)^d^	(36.1±0.4)^d^	(16.9±1.1)^e^
				18		(29.6±1.3)^de^		(45.9±0.6)^e^	(28.9±0.4)^f^	(37.6±0.2)^b^
10		0		6		(38.2±1.3)^bc^		(54.9±0.6)^b^	(45.9±0.4)^b^	(11.3±0.1)^f^
				12		(28.1±0.6)^e^		(50.4±0.9)^cd^	(38.2±0.2)^d^	(14.6±0.2)^ef^
				18		(20.3±0.9)^f^		(45.3±0.3)^e^	(30.7±0.4)^ef^	(31.9±0.1)^c^
5		0.1		6		(52.6±0.6)^ef^		(60.2±1.5)^a^	(57.4±1.6)^a^	(9.5±0.2)^f^
				12		(48.2±0.6)^a^		(55.6±0.4)^b^	(44.9±0.9)^b^	(11.4±0.1)^f^
				18		(39.1±0.6) ^bc^		(53.1±0.6)^bc^	(32.1±0.3)^e^	(22.7±0.3)^d^
Significant				df						
Time				2		**		**	**	**
Treatment				3		**		**	**	**
T×T				6		ns		**	**	**
CV				-		7.39		2.15	2.92	7.52

Data are presented as mean value±standard error of three replications. Different letters indicate significant differences at p=0.05. * and **Significance at p=0.05 and 0.01, respectively. GAE=galllic acid equivalents, PAL=phenylalanine ammonia-lyase, PPO=polyphenol oxidase

In walnuts, light kernel colour is crucial for sensory quality attributes leading to consumer preference. According to Christopoulos and Tsantili (*27*), phenolics are accumulated in walnut kernels by the activation of phenylpropanoid pathway through PAL enzyme activity, which is responsible for non-oxidative deamination of phenylalanine to *trans*-cinnamic acid. Walnut kernel phenolics have a high antioxidant capacity, which contributes to a higher DPPH or FRAP scavenging capacity. Phenolics are crucial for the human health and play a crucial role in attenuating oxidative UNSFA rancidity in the walnut kernels by obstructing ROS accumulation (*28*, *29*). However, under unfavourable postharvest handlings and due to membrane integrity damage caused by MDA accumulation, higher phenolic accumulation in walnut kernels may contribute to surface browning in kernels, as a result of the increased PPO (enzyme responsible for browning) activity, which leads to a lower consumer acceptability and reduced sensory quality (*3*, *10*, *30*). In line with oxidative rancidity, kernel surface browning as a consequence of phenolic oxidation by PPO results in a huge reduction in kernel antioxidant capacity (*31*). Fuentealba *et al*. (*32*) reported that the walnut kernel light colour was accompanied with higher phenolic accumulation resulting from lower phenolic oxidation by PPO activity and seemingly attributed higher DPPH scavenging capacity. Likewise, light colour kernels exhibit a higher arbutin accumulation and antioxidant activity, which is crucial for attenuating the browning incidence by hampering tyrosinase enzyme activity. Along with higher phenolic accumulation, light walnut colour was accompanied with higher linolenic acid accumulation, revealing the lessening of oxidative rancidity *via* antioxidant phenolics (*32*). Moreover, Wang *et al*. (*4*) reported that the green hull of fresh walnuts in the hull packed in modified atmosphere packaging during storage at 1 °C for 60 days exhibited higher phenolic and flavonoid accumulation, leading to higher FRAP scavenging capacity derived from higher PAL/PPO enzyme activity, which diminished the decay of fresh walnut in the hull and maintained kernel quality with lower PV (*4*). Christopoulos and Tsantili (*31*) reported that the walnut kernels stored at 20 ºC for 9 months exhibited a higher surface browning, reflected by the lower L*, *h*º and WI, along with lower FRAP and DPPH˙ scavenging capacity due to phenolic oxidation. In comparison, walnut kernels stored at low temperature (1 °C) and packed under N_2_ or CO_2_ exhibited a lower kernel surface browning and higher FRAP and DPPH˙ scavenging capacity owing to the increased phenolic accumulation, as the consequence of reduced phenolic oxidation by the low temperature and/or low O_2_ availability (*30*). Christopoulos and Tsantili, (*31*) suggested that walnut kernel surface browning at a higher temperature and O_2_ availability may be attributed to lower FRAP and DPPH˙ scavenging capacity and the lower phenolic accumulation caused by the higher phenolic oxidation. Based on the data from the present experiment, to overcome the oxidative browning in walnut kernels, coating with gum arabic (5%) enriched with GABA (0.1 mM) would be an applicable method due to higher PAL/PPO enzyme activity concurrent with higher phenolic accumulation and higher DPPH˙ scavenging capacity, mainly due to the synergistic behaviour of gum arabic coating function through lowering O_2_ availability with the ROS scavenging capacity of GABA. In addition to phenolics, GABA itself exhibits a strong ROS scavenging activity and may contribute to reduction of oxidative browning of walnut kernels (*25*).

## CONCLUSIONS

In conclusion, the present study sheds light on the beneficial impacts of gum arabic coating enriched with γ-aminobutyric acid (GABA) on attenuating the oxidative rancidity and browning of walnut kernels stored at 20 °C for 18 weeks. The results of this study demonstrate that coating of nuts with 5% gum arabic enriched with GABA (0.1 mM) could control kernel lipid oxidation and undesirable phenolic changes during storage at 20 °C, as compared to uncoated walnut kernels. The reduction of oxidative rancidity and browning of walnut kernels by gum arabic coating enriched with GABA may be the result of lower lipoxygenase activity along with lower H_2_O_2_ accumulation, giving rise to higher UNSFA/SFA ratio, and of higher PAL/PPO enzyme activity, giving rise to higher phenolic accumulation and higher DPPH˙ scavenging capacity. Taken together, synergistic action of gum arabic coating, which lowers O_2_ availability, and the antioxidant function of GABA may be responsible for the reduction of oxidative rancidity and browning of walnut kernels.
